# Risk Factors of Saphenous Vein Insufficiency in Female Patients in Riyadh, Saudi Arabia

**DOI:** 10.7759/cureus.6643

**Published:** 2020-01-13

**Authors:** Abdullah Alwahbi, Amal Alamri, Wafa Alotaibi

**Affiliations:** 1 Vascular Surgery, King Abdulaziz Medical City, Ministry of National Guard Health Affairs, Riyadh, SAU; 2 Vascular Surgery, King Saud Bin Abdulaziz University for Health Sciences, Riyadh, SAU; 3 Vascular Surgery, King Saud Bin Abdulaziz University for Health Sciences (ksau-Hs), Riyadh, Saudi Arabia, Riyadh, SAU

**Keywords:** saphenous vein, venous insufficiency, retrospective, female population, saudi arabia

## Abstract

Background: Venous reflux most frequently occurs in the great saphenous vein (GSV), which is the most commonly diseased vein and is associated with venous insufficiency and varicose veins.

Objective: We aimed, in this study, to determine risk factors for saphenous vein insufficiency in female patients in Riyadh, Saudi Arabia.

Methods: This was a retrospective cohort study. Data were collected from the medical records of adult female patients who developed saphenous vein insufficiency at King Abdulaziz Medical City, between 2015 and 2017.

Results: A total of 97 subjects participated in the study, 53 patients had saphenous vein reflux while 44 patients did not have reflux. Leg swelling was the only symptom that showed a significant difference between the two groups (p<0.001); patients with reflux had significantly higher rates of endovenous laser therapy (p=0.021). While the same patient group showed significantly lower rates of sclerotherapy compared to patients without reflux (p=0.006). The proportion of hypertension patients without reflux (22.7%) was significantly larger than that of hypertension patients with reflux (1.9%) (p= 0.001).

Conclusion: More research is encouraged to provide better understanding and management of saphenous vein insufficiency in the Saudi population, especially in females who are at high risk.

## Introduction

Lower limb saphenous vein insufficiency is a frequent medical disorder which is detected in about 30% of adult females and about 15% of males in the European community [1]. Most of the patients with saphenous vein insufficiency have an involvement of the great saphenous vein (GSV) followed by the small saphenous vein [2-3]. 

Traditional clinical manifestations symptoms and signs of saphenous vein insufficiency are edema, discomfort, aching, in addition to muscle cramps [4]. Some complications may occur with saphenous vein insufficiency comprising venous ulcerations, eczema, and superficial thrombophlebitis [5-7].

Chronic venous insufficiency (CVI) can have a large influence on the quality of life of patients that can be compared to other frequent disorders [8]. It is also linked to increased healthcare costs. The treatment of saphenous vein insufficiency decreases the symptoms as well as complications. Moreover, it improves health-related quality of life [9].

Turning to management modalities, during the past few years, endogenous laser ablation therapy of saphenous veins has shown to be safe. Additionally, long-term follow up showed comparable results that were sometimes superior to conventional surgical strategies [10-11]. However, the success of this relatively novel technique is limited by some post-procedure complications like post-procedure pain, phlebitis, and tenderness, for instance [12-14]. Yet, data on risk factors of saphenous vein insufficiency is scarce and confusing in medical literature, especially in the gulf area and Arab region [15].

Since data regarding saphenous vein insufficiency are lacking, this retrospective cohort study aimed to determine risk factors associated with saphenous vein insufficiency in female patients in Riyadh, Saudi Arabia.

## Materials and methods

This was a retrospective cohort study conducted to determine risk factors associated with saphenous vein insufficiency in female patients in Riyadh, Saudi Arabia. Data were collected from the medical records of the female patients who developed saphenous vein insufficiency at the King Abdulaziz Medical City hospital between 2015 and 2017. Data were collected from charts, Best-care systems were then recorded in data collection forms and finally entered into excel spreadsheets.

In addition to the patients’ characteristics (age, weight, height, body mass index (BMI)), previous pregnancies, symptoms of saphenous vein insufficiency, previous treatment of varicose veins, and risk factors of saphenous vein insufficiency were collected. Symptoms collected included leg pain, leg swelling, leg lymphedema, and skin pigmentation. Previous treatment of saphenous vein insufficiency included previous vein surgery, previous endovenous laser therapy, and previous sclerotherapy. Risk factors included diabetes mellitus, hypertension, high lipid levels, smoking, heart disease, field work, oral contraceptive pills, and family history of varicosities. Participation in the study was voluntary, and every respondent provided written informed consent. Participants were not asked to provide their names, and confidentiality and anonymity were maintained throughout the study. Only the research team had access to the data

Statistical analyses

Data were represented in terms of frequencies (number of patients/ cases) and valid percentages for categorical variables. Mean, standard deviations (SD), minimum and maximum values were used to describe the numerical variable. Chi-square test was used to compare categorical variables between the subgroups (cross-tabulation) while one-way Anova test was used to compare numerical variables between the subgroups. All P values < 0.05 were considered statistically significant. IBM Statistical Package for the Social Science (SPSS; IBM Corp, Armonk, NY) version 21 for Microsoft Windows was used to perform all statistical calculations.

## Results

The following patients’ characteristics were collected:

Age

The mean± SD age of participating females was 43± 12.7 years with a minimum value of 21 and a maximum value of 74 years.

Physical examination

The mean weight was 77.7± 13.9 kg with a minimum value of 53 kg and a maximum value of 136 kg, the mean height was 159.5± 5.2 cm with a minimum value of 143 cm and a maximum value of 173 cm, and the mean body mass index (BMI) was 30.7± 6 kg/m2 with a minimum value of 19.9 kg/m2 and a maximum value of 55.9 kg/m2.

Previous pregnancies

Regarding the number of previous pregnancies, we found that 22.7% of patients (n= 20) had a total of three pregnancies, 17% had a total of four pregnancies, 17% had a total of two pregnancies, and 15.9% had a total of five pregnancies.

Symptoms of venous insufficiency

We found that the vast majority (n= 96, 99%) had venous insufficiency symptoms; 96 (99%) patients suffered from leg pain, 77 (79.4%) patients experienced leg swelling, only four (4.1%) had leg lymphedema, and only one (1%) experienced skin pigmentation. Additionally, symptoms were compared with patients who had saphenous reflux and those who did not have saphenous reflux using the chi-square test; P value ≤0.05 was considered significant. There was a significantly higher (P<0.001) rate of leg swelling in patients with reflux (100%) compared to patients without reflux (54.45%). However, leg pain, leg lymphedema, and skin pigmentation did not differ in the two groups (Table 1).

**Table 1:** Symptoms of venous insufficiency reported in the study population *Level of significance at P value ≤0.05

Symptom	Yes/ No	With Reflux (N=53)	Without Reflux (N=44)	P Value
Leg pain	Yes	53 (100)	43 (97.7)	0.270
	No	0	1 (2.27)	
Leg swelling	Yes	53 (100)	24 (54.45)	<0.001*
	No	0	20 (45.45)	
Leg lymphedema	Yes	4 (7.54)	0	0.063
	No	49 (92.45)	44 (100)	
Skin pigmentation	Yes	1 (1.88)	0	0.360

Previous treatment of venous insufficiency

It was found that the vast majority (n= 96, 99%) had no previous vein surgery, while only one (1%) patient underwent the surgery. Only six (6.2%) patients had previous endovenous laser therapy, and only nine (9.3%) patients were found to have previous sclerotherapy. Furthermore, patients with reflux were compared to patients without reflux using the chi-square test. It was observed that patients with reflux had significantly higher rates of previous endovenous laser therapy (P=0.02). On the other hand, patients without reflux had significantly higher rates of sclerotherapy (P=0.006) (Table 2).

**Table 2:** Previous treatment of venous insufficiency among the study population *Level of significance at P value ≤0.05

Previous treatment	Yes/ No	With Reflux (N=53)	Without Reflux (N=44)	P value
Previous endovenous laser therapy	Yes	6 (11.32)	0	0.021*
	No	47 (88.67)	44 (100)	
Previous sclerotherapy	Yes	1 (1.88)	8 (18.18)	0.006*

Risk factors for saphenous vein insufficiency

Out of the 97 included patients, we found that 21 (21.6%) patients had diabetes mellitus, 11 (11.35%) patients have hypertension, and 15 (15.5%) patients have high lipid levels. All of the included patients (n= 97, 100%) were found to be non-smokers. Also, they did not have heart diseases (n= 97, 100%). In regards to varicosities, 40 (41.2%) patients were found to have a family history of varicosities. As for field work, 29 (29.9%) patients were found to have field work. The majority (n= 79, 81.4%) were found to be receiving oral contraceptive pills, and we also found that 46 (52.2%) patients had less than or equal to three pregnancies. Also, the risk factors in both patients with reflux and those without reflux were compared. It was observed that hypertension was significantly higher (p=0.001) in patients without reflux. However, previous pregnancies and field work were higher in patients with reflux with a P value 0.018 and <0.033, respectively.

Reflux status

A total of 97 (100%) female patients were included in this study from King Abdulaziz Medical City in Riyadh, Saudi Arabia out of which, 53 (54.6%) patients were suffering from saphenous reflux while 44 (45.4%) patients had no saphenous reflux.

Determination of risk factors associated with saphenous vein insufficiency (with or without saphenous reflux)

Our results showed that patients without reflux were older than patients with reflux; however, the difference was insignificant (p= 0.565). In terms of BMI, patients with reflux and patients without reflux had a mean BMI of 30.7± 11.7 kg/m2 and 30.8± 6.6 kg/m2. The difference was also insignificant between the two groups (p= 0.935). Regarding diabetes mellitus, we found that 12 (27.3%) diabetes mellitus patients were without reflux while nine (17.0%) diabetes mellitus patients were with reflux. The difference was also insignificant (p= 0.164). Similar findings were observed concerning high lipid levels; it was found that the proportion of patients without reflux who had high lipid levels (n=10, 22.7%) was insignificantly larger than that of patients with reflux who had high lipid levels (n= 5, 9.4%), (p= 0.064). A statistically significant difference (p= 0.001) was found between hypertension patients without reflux and those with reflux. Interestingly, the proportion of hypertension patients without reflux (n= 10, 22.7%) was larger than that of hypertension patients with reflux (n= 1, 1.9%). In regard to field work, a statistically significant difference (p= 0.018) was observed between the two groups (with & without reflux). We found that the proportion of field workers who had reflux (n= 21, 39.6%) was larger than that of field workers who had no reflux (n= 8, 18.2%). The same pattern was observed concerning previous pregnancies (more than three); we found that the proportion of patients with reflux who had more than three previous pregnancies (n= 30, 56.6%) which was larger than that of patients without reflux who had more than three previous pregnancies (n= 12, 27.3%). A statistically significant difference was observed between the two groups (p= 0.033). Conversely, there were no statistically significant differences between the two groups in terms of using oral contraception pills and family history of varicosities, (p=0.366 and p=0.395 respectively) (Table 3).

**Table 3:** Risk factors for venous insufficiency with and without saphenous reflux *Level of significance at P value ≤0.05

Risk factors	With reflux Mean± SD / Count (%)	Without reflux Mean± SD / Count (%)	P value*
Age (years)	42.3± 11.7	43.8± 13.9	0.565
BMI (kg/m^2^)	30.7± 5.6	30.8± 6.6	0.935
Diabetes mellitus	9 (17.0%)	12 (27.3%)	0.164
Hypertension	1 (1.9%)	10 (22.7%)	0.001*
High lipid levels	5 (9.4%)	10 (22.7%)	0.064
Field work	21 (39.6%)	8 (18.2%)	0.018*
Oral contraceptive pills	11 (20.8%)	7 (15.9%)	0.366
Family history of varicosities	23 (43.4%)	17 (38.6%)	0.395
Previous pregnancies (More than 3)	30 (56.6%)	12 (27.3%)	0.033*
Smoking	Not applicable, as all patients are non-smokers		
Heart diseases	Not applicable, as all patients have no heart diseases		
*One way Anova test was used to compare between the different subgroups			

## Discussion

Chronic venous disease (CVD) comprises a wide range of chronic conditions that are related to or caused by veins, such as telangiectases, varicose veins, CVI especially in the saphenous vein, phlebitis, skin hyperpigmentation, and ulcer formation [16].

CVI is considered to be among the most common manifestations of CVD. CVI accounts for a condition that affects the venous system of the lower limbs. CVI often refers to the more progressive forms of venous disorders, including skin hyperpigmentation, venous eczema, and ulcers. Nevertheless, because incompetent valves and increased venous pressure are also involved in varicose veins, the term CVI is used to represent the full spectrum of manifestations of CVD [6,17].

The clinical, aetiological, anatomical, and pathological (CEAP) classification system was created in order to standardize the reporting and treatment of the diverse manifestations of CVD. The revised version of the classification additionally contains definitions of clinical signs and suggests three levels of investigations adjusted to the clinical stage [12,18]. Both the basic CEAP and comprehensive CEAP were created to allow uniform diagnosis and comparison of different patient populations [18].

The GSV and the short saphenous vein (SSV), along with perforator veins (PV) are the most commonly affected veins by abnormal valve function [19]. The GSV, the longest vein in the human body, is a superficial vein that extends from the dorsum of the foot to the upper thigh and groin. The significance of GSV comes from its role in returning blood from the leg to the heart and is also used in several major medical procedures [20]. Since data about CVI, including CVD and saphenous vein insufficiency, in the female population in Saudi Arabia are still lacking, our current study aimed to get a better understanding about the underlying disease, and also to determine risk factors associated with saphenous vein insufficiency in female patients in Riyadh, Saudi Arabia.

Our results showed that 54.6% of the total population was suffering from saphenous reflux. Two studies with only female populations, that were conducted in 2005 and 2007, elaborated related information that GSV reflux was present in 60%, and 39%, respectively, of the total extremities examined. SSV reflux was found to be 3%, and 2%, respectively, while both GSV and SSV reflux were found to be present in 17%, and 5%, respectively, of the total extremities examined [21-22].

Over the years, numerous studies have investigated the prevalence of CVI, especially saphenous vein insufficiency, and the associated risk factors. The results vary according to the geographic location and the population being studied [7-9]. The Edinburgh Vein Study reported that the prevalence of all categories of varicose veins and CVI increased with age (p < 0.001), while no relation was found with social class [15]. The same study investigated the lifestyle risk factors in another paper. It reported that obesity, previous pregnancy, lower use of oral contraceptive pills, and mobility at work may be implicated in venous reflux in women, while in men, height, and straining at stool may be implicated as risk factors [23].

In the current study, we found that field work and previous pregnancies (more than three) are significantly associated with saphenous reflux (p= 0.018 & p= 0.033, respectively). On the other hand, diabetes mellitus, high lipid levels, taking oral contraception pills, and family history of varicosities were found to have no association with saphenous reflux. Unexpectedly, the proportion of hypertension patients without reflux (22.7%) was significantly larger than that of hypertension patients with reflux (1.9%) (p= 0.001). This may be due to the very small number of hypertensive patients with reflux (only one patient). In the current study, we found that field work and previous pregnancies (more than three) are significantly associated with saphenous reflux (p= 0.018 & p= 0.033, respectively).

Turning to the treatment method, patients with reflux had significantly higher rates of endovenous layer therapy (p=0.021). While the same patient group showed significantly lower rates of sclerotherapy compared to patients without reflux (p=0.006). Results from Edinburgh Vein Study showed that the prevalence of all categories of varicose veins and CVI increased with age (p< 0.001), while no relation was found with social class [15]. It was also reported that obesity, previous pregnancy, lower use of oral contraceptive pills, mobility at work may be implicated in venous reflux in women [23]. In alignment with the Edinburgh Vein Study, constipation was also found to be one of the lifestyle risk factors associated with CVI [23-24]. Another study showed that obesity, lack of physical activity, and the number of previous pregnancies were significantly associated with CVI in women [25-26].

We recommend conducting further prospective studies preferably with a larger sample size to get a more accurate estimation of the prevalence of saphenous venous insufficiency and better determine the potential risk factors in our specific population, especially adult females with high risk.

## Conclusions

We conclude that leg pain and leg swelling were found to be the most common symptoms of saphenous vein insufficiency. In addition, field work and high frequency of previous pregnancies may be associated with a higher risk of developing saphenous vein insufficiency with reflux (p=0.018 and p=0.033, respectively). Therefore, it is very important to raise the awareness of healthcare providers and female patients about this disease, its symptoms, and the preventable risk factors in order to minimize its prevalence and ensure a better quality of life for Saudi females.
